# Antibacterial and antibiotic resistance modulatory activities of leaves and bark extracts of *Recinodindron heudelotii* (Euphorbiaceae) against multidrug-resistant Gram-negative bacteria

**DOI:** 10.1186/s12906-017-1687-2

**Published:** 2017-03-24

**Authors:** Aimé Gabriel Fankam, Jules-Roger Kuiate, Victor Kuete

**Affiliations:** Department of Biochemistry, Faculty of science, University of Dschang, P.O. Box 67, Dschang, Cameroon

**Keywords:** *Recinodindron heudelotii,* Gram-negative bacteria, MDR bacteria, antibacterial activity, antibiotic modulators

## Abstract

**Background:**

*Recinodindron heudelotii* (Euphorbiaceae) is a plant used in Africa, particularly in Cameroon to treat various ailments including bacterial infections. In this study, we evaluated the extracts of the leaves (RHL) and bark (RHB) of *R. heudelotii* for their antibacterial and antibiotic resistance modulating activities against 29 Gram-negative bacteria, including multidrug-resistant (MDR) phenotypes.

**Methods:**

The broth micro-dilution assay was used to evaluate the antibacterial activity, and the antibiotic resistance modulating effects of the plant extracts.

**Results:**

RHL displayed the most important spectrum of activity with minimal inhibitory concentrations **(**MICs) values ranging from 256 to 1024 μg/mL against 75.86% of the 29 tested bacteria strains while RHB was not active. RHL also showed killing effects with minimal bactericidal concentrations (MBCs) ranging from 256 to 1024 μg/mL. The activities of tetracycline and kanamycin associated with RHL were improved on 88.89% and 77.78% of the tested MDR bacteria, at MIC/2 at MIC/4 respectively, with 2 to 16-folds decreasing of MIC. This suggests the antibiotic resistance modulating effects of these antibiotics.

**Conclusion:**

The present study provides data indicating a possible use of the leaves extract of *Recinodindron heudelotii* alone or in association with common antibiotics in the fight against bacterial infections including those involving MDR bacteria.

## Background

Infectious diseases caused by multidrug-resistant (MDR) bacteria constitute nowadays a real public health concern all over the world. These bacteria drastically reduced the efficacy of antibiotic arsenal, consequently, increasing the frequency of therapeutic failure and mortality [1, 2]. In the European Union, it was estimated that 25,000 patients die annually due to infections with MDR bacteria [3]. Among these MDR bacteria, Gram-negative MDR bacteria drastically impair the efficacy of antibiotic families and consequently limit their clinical uses [4, 5]. Due to these facts, scientists are in the quest for new antimicrobial substances. Nature is a source of medicinal agents since times immemorial. The screening of plant extracts and natural products for their antimicrobial activity has shown that higher plants represent a potential source of novel antibiotic prototypes [6–8]. In the last decades, a number of studies conducted, demonstrated that plants as well as their derived products could possess direct antimicrobial activity and resistance modifying effects [9–14]. Thus, with increased incidence of resistance to antibiotics, natural products from plants could be an interesting alternative.

*Recinodindron heudelotii* (Baill.) Pierre ex Pax. known as “Djansang or Essessang” in different area in Cameroon, is a tree belonging to Euphorbiaceae family. With 20–30 m of height, that plant grows throughout the humid lowland rainforest of Cameroon [15, 16]. This plant is traditionally used in Cameroon and certain countries in Africa to treat cough, intestinal disease, dysentery and as antidote [15, 17]. Furthermore, *R. heudelotii* is also used to ease delivery, treat diseases such as malaria, anaemia, stomach pain, yellow fever and as aphrodisiac. Its seeds are also used as food ingredient [16]. *R. heudelotii* is well documented for some pharmacological properties among which antimicrobial [18, 19] and antioxidant activities [20]. In the continuous search for antibacterial agents from that plant, we have designed this study to investigate the in vitro antibacterial and antibiotic resistance modulating activities of the methanol extracts from leaves and stem bark of *R. heudelotii* against MDR Gram-negative bacteria.

## Methods

### Plant materials and extraction

The leaves and bark of *R. heudelotii* were collected in April 2012 at Melong, Littoral-Cameroon. The plant was identified at the National Herbarium in Yaoundé (Cameroon), by a botanist under the registration number 19695 SRF/Cam. The dried and powdered material (100 g) of each plant was macerated in 300 mL of methanol for 48 h at room temperature, and then filtered using Whatman filter paper No.1. The filtrate obtained was concentrated using a rotary evaporator under reduced pressure to obtain the extracts, which were kept at 4 °C until usage.

### Chemicals for antibacterial assays

Six antibiotics: tetracycline (TET), kanamycin (KAN), erythromycin (ERY), ciprofloxacin (CIP), chloramphenicol (CHL) and ampicillin (AMP) (Sigma-Aldrich, St Quentin Fallavier, France) were used. *p*-Iodonitrotetrazolium chloride 0.2% (INT) (Sigma-Aldrich) was used as bacterial growth indicator and dimethyl-sulfoxide (DMSO) was used to dissolve the extracts.

### Bacteria strains used and growth conditions

The microbial species used in the study were Gram-negative bacteria including MDR and reference strains of *Escherichia coli, Enterobacter aerogenes, Klebsiella pneumoniae, Enterobacter cloacae, Pseudomonas aeruginosa,* and *Providencia stuartii*. Their features were previously reported [14, 21]. All strains were cultured overnight on Mueller Hinton Agar 24 h prior to any assay. The Mueller Hinton Broth (MHB) was used as liquid culture medium for susceptibility tests.

### Evaluation of the antibacterial activity

The antibacterial activities of the extracts were determined using rapid INT colorimetric assay [22, 23]. Two fold serial dilution of the extract (dissolved in DMSO/MHB) were made in a 96-well microplate. Then, 100 μL of inoculums (1.5× 10^6^ CFU/mL) prepared in MHB were then added. The plates were covered with a sterile plate sealer and then agitated with a shaker to mix the contents of the wells and incubated at 37 °C for 18 h. Wells containing MHB, 100 μl of inoculums and DMSO at a final concentration of 2.5% served as negative control (This internal control was systematically added). The minimal inhibitory concentration (MIC), defined as the lowest sample concentration that prevented the growth of the bacteria was then detected after the addition of 40 μL of INT (0.2 mg/mL) in each well of the plates and incubated at 37 °C for 30 min. The minimal bactericidal concentration (MBC) of each sample was determined by adding 50 μL aliquots of the preparations which did not show any growth after incubation during MIC determination to 150 μL of MHB. These preparations were incubated at 37 °C for 48 h. MBC was regarded as the lowest concentration of sample that prevented the colour change of the medium after addition of INT as mentioned above [23].

### Modulation assays

To evaluate the extracts of *R. heudelotii* as a modulator of antibiotic resistance, MICs of antibiotics were determined in the absence and presence of these extracts using the broth micro-dilution method as previously described [24, 25]. Briefly, after serial dilutions of antibiotics (0.5–256 휇g/mL), the most active extract; RHL, was added to each well at its sub-inhibitory concentrations and the inoculation was done. The MIC was determined as described above. Rows receiving antibiotic dilutions without extracts were used for the determination of the MICs of the antibiotics. The modulation factor was defined as the ratio of the MIC for the antibiotic alone and that of the antibiotics in the presence of the extract (RHL). Modulation factor ≥ 2 was set as the cut-off for biologically significance of antibiotic resistance modulating effects [11].

## Results

### Antibacterial activity of the plant extracts

The data summarized in Table 1 show that extracts tested were active on at least two bacteria with MIC values varying from 256 to 1024 μg/mL. Leaves extract of *R. heudelotii* (RHL) displayed the most important spectrum of activity with MICs ranging from 256 to 1024 μg/mL against 75.86% of the tested bacteria while its bark extract (RHB) was active only on tree bacterial strains. In general, RHL showed MICs below 625 μg/mL against 44.83% of the 29 tested bacteria. It’s lowest MIC (256 μg/mL) has been recorded on *E. coli* W3110 and *Enterobacter aerogenes* EA3. Chloramphenicol showed variable inhibitory activities depending on bacteria strains, with MICs below 10 μg/mL against 31.03% (9/29) of the tested bacteria. RHL also presented MBCs ranging from 256 to 1024 μg/mL.

**Table 1:** Antibacterial activity of *Recinodindron heudelotii* against selected Gram-negative bacteria

Bacteria used	Tests samples, MIC and MBC (μg/mL)					
	*Recinodindron heudelotii*				CHL	
	Leaves (RHL)		Bark (RHB)			
	MIC	MBC	MIC	MBC	MIC	MBC
*E. coli*						
ATCC8739	512	-	-	-	2	32
ATCC10536	1024	1024	-	-	1	32
AG100	1024	-	-	-	8	128
AG100A	512	1024	-	-	0,5	64
AG100A_Tet_	1024	-	-	-	64	256
AG102	1024	-	-	-	32	-
MC4100	512	-	1024	-	32	-
W3110	256	-			8	128
*E. aerogenes*						
ATCC13048	1024	-			8	64
CM64	1024	-			-	-
EA27	512	1024	1024	-	64	256
EA3	256	256	-	-	128	-
EA289	1024	-	-	-	128	-
EA298	-	-	-	-	64	-
EA294	512	1024	512	-	16	64
*E. cloacae*						
ECCI69	-	-	-		-	-
BM47	-	-	-		-	-
BM67	-	-	-		-	-
*K. pneumoniae*						
ATCC11296	1024	-	-	-	4	32
KP55	512	1024	-	-	64	256
KP63	512	1024	-	-	64	256
K24	-	-	-	-	32	256
K2	512	512	-	-	16	256
*P. stuartii*						
ATCC29916	512	-	-	-	8	128
NEA16	-	-	-	-	64	256
PS2636	512	-	-	-	32	256
PS299645	512	-	-	-	32	256
*P. aeruginosa*						
PA01	1024	-	-	-	32	256
PA124	-	-	-	-	64	-

*MIC* Minimal Inhibitory Concentration, *MBC* Minimal Bactericidal Concentration, *CHL* Chloramphenicol **-**: MIC and MBC not detected at up to 1024 μg/mL for plant extracts and 256 μg/mL for reference drug (CHL)

### Antibiotic resistance modulating effects of the plant extracts

The results of the pre-screening of the *R. heudelotii* extracts for their resistance modulating effects (Table 2) against MDR *P. aeruginosa* PA124, allowed us to select the leaves extract of *R. heudelotii* (RHL) at its sub-inhibitory concentrations (half and quarter of MIC) for the study of its antibiotic resistance modulating effects against selected MDR Gram-negative bacteria. Table 3 shows the antibacterial activity of six commonly used antibiotics in the presence of RHL against selected MDR Gram-negative bacteria. RHL has significantly improved the activity of TET, KAN and CHL against most of the tested bacteria at it sub-inhibitory concentrations. At MIC/2, it modulated in more than 70% of cases, the activity of TET and KAN (88.89%) and in 66.67% those of CHL. At MIC/4, RHL showed important modulating effects with TET and KAN, respectively on 77.78% and 66.67% of the tested bacteria. No modulating effect was noted with ampicillin in the presence of that extract.

**Table 2:** Antibiotic resistance modulatory activity of *R. heudelotii* at sub-inhibitory concentrations against *P. aeruginosa* PA124

Plant extracts^a^	Extract concentrations^b^	Antibiotics^c^ and minimal inhibitory concentration (μg/mL)					
		CHL	AMP	ERY	KAN	TET	CIP
	0	64	-	128	128	16	32
RHL	MIC/2	32 (**2**)	-	64(**2**)	64 (**2**)	4 (**4**)	32 (1)
	MIC/4	32 (**2**)	-	128 (1)	64 (**2**)	8 (**2**)	32 (1)
	MIC/8	32 (**2**)	-	128 (1)	64 (1)	8 (**2**)	32 (1)
	MIC/16	64 (1)	-	128 (1)	64 (1)	16 (1)	32 (1)
RHB	MIC/2	64 (1)	-	128 (1)	128 (1)	8 (**2**)	32 (1)
	MIC/4	64 (1)	-	128 (1)	128 (1)	16 (1)	32 (1)
	MIC/8	64 (1)	-	128 (1)	128 (1)	16 (1)	32 (1)
	MIC/16	64 (1)	-	128 (1)	128 (1)	16 (1)	32 (1)

^a^: *Recinodindronheudelotii*, leaves (RHL); barks (RHB).^b^ *: MIC* Minimal Inhibitory Concentration ^c^: *TET* tetracycline, *KAN* kanamycin,, *ERY* erythromycin, *CHL* chloramphenicol, *CIP* ciprofloxacin, *AMP* ampicillin; −: MIC not detected at up to 256 μg/mL; (): Modulating factor; Values in bold represent the modulating factor ≥ 2

**Table 3:** Antibiotic resistance modulatory activity of leaves extract of *R. heudelotii*

Antibiotics^a^	Extracts concentration^b^	Bacteria, MIC (μg/mL) and modulating factors (in bracket)									Modulating effect (%)^b^
		*E. coli*			*E. aerogenes*			*K. pneumoniae*	*P. stuartuii*	*P. aeruginosa*	
		AG100	AG102	AG100_Tet_	CM64	EA289	EA298	K24	NEA16	PA124	
AMP	0	-	-	-	-	-	-	-	-	-	
	MIC/2	-	-	-	-	-	-	-	-	-	0
	MIC/4	-	-	-	-	-	-	-	-	-	0
CHL	0	8	32	64	256	128	64	64	64	64	
	MIC/2	8 (1)	16 (**2**)	32 (**2**)	256 (1)	128 (1)	32 (**2**)	32 (**2**)	32 (**2**)	32 (**2**)	66.67
	MIC/4	8 (1)	16 (**2**)	64 (1)	256 (1)	256 (0.5)	64 (1)	64 (1)	32 (**2**)	32 (**2**)	33.33
CIP	0	≤0.5	≤0.5	128	64	128	1	128	1	32	
	MIC/2	≤0.5 (na)	≤0.5 (na)	64(**2**)	32 (**2**)	128 (1)	1 (1)	64 (**2**)	1 (1)	32 (1)	33.33
	MIC/4	≤0.5 (na)	≤0.5 (na)	128(1)	64 (1)	128 (1)	1 (1)	64 (**2**)	1 (1)	32 (1)	11.11
KAN	0	64	4	8	2	16	32	4	16	128	
	MIC/2	4 (**16**)	1 (**4**)	2 (**4**)	1 (**2**)	4 (**4**)	32 (1)	2 (**2**)	8 (**2**)	64 (**2**)	**88.89**
	MIC/4	16 (**4**)	4 (1)	2 (**4**)	2 (1)	8 (**2**)	32 (1)	2 (**2**)	8 (**2**)	64 (**2**)	66.67
ERY	0	32	64	-	-	64	16	128	16	128	
	MIC/2	32 (1)	64 (1)	-	-	64 (1)	16 (1)	128 (1)	8 (**2**)	64 (**2**)	22.22
	MIC/4	32 (1)	64 (1)	-	-	64 (1)	16 (1)	128 (1)	8 (**2**)	128 (1)	11.11
TET	0	2	8	32	16	32	4	16	4	16	
	MIC/2	≤0.5 (**≥4**)	4 (**2**)	4 (**8**)	4 (**4**)	4 (**8**)	2 (**2**)	8 (**2**)	2 (**2**)	16 (1)	**88.89**
	MIC/4	≤0.5 (**≥4**)	4 (**2**)	8 (**4**)	16 (1)	8 (**4**)	2 (**2**)	8 (**2**)	2 (**2**)	16 (1)	**77.78**

^a^: *TET* tetracycline, *KAN* kanamycin, *ERY* erythromycin, *CHL* chloramphenicol, *CIP* ciprofloxacin, *AMP* ampicillin;*;* −: MIC not detected at up to 256 μg/mL; (): Modulating factor; na: not applicable,

^b^: *MIC* Minimal Inhibitory Concentration. ^c^: Percentage of antibiotic’s modulating effect by the plant extracts. Values in bold represent modulating factor ≥ 2 and modulating effect observed on more than 70% of the tested MDR bacteria

## Discussion

The objective of this study was to evaluate the methanol extracts from the leaves (RHL) and bark (RHB) of *R. heudelotii* for their antibacterial and antibiotic resistance modulating activities against a panel of Gram-negative bacteria including multidrug resistant (MDR) phenotypes. For phytochemical agents, MICs ranging from 100 to 1000 μg/mL obtained after susceptibility tests indicate their antimicrobial activities [26]. Thus, leaves extract of *R. heudelotii* (RHL) displayed antibacterial activity against 75.86% of the tested bacteria whereas its bark extract (RHB) was not active. According to the cut-off value of MICs for extracts as proposed by Kuete [8], RHL presents moderate antibacterial activity (100 ≤ MIC ≤625 μg/mL) against the tested bacteria. A keen look the MICs and MBCs of RHL (Table 1) indicated that it possesses bactericidal or killing effects (MBC/MIC ≤4) [27]. *R. heudelotii* bark extract was previously documented for its low antimicrobial activity against some pathogenic bacteria including Gram-negative and Gram-negative bacteria [18, 19]. This confirms low antibacterial activity observed with its bark methanol extract (RHB) in the present study. By considering the MDR features of the tested bacteria, RHL could be a source for the development of new antibacterial agents.

The antibiotic resistance modulating effects of the extracts or natural compounds from medicinal plants against resistant bacteria have been already reported [11, 13, 28–30]. In this work, the combination of extracts with antibiotics has shown that extract of the leaves of *R. heudelotii* (RHL) modulated 2 to 16 folds the activity of TET, KAN and CHL against selected MDR bacteria. Based on the previous work, one of the mechanisms of action of plant extracts associated with antibiotics may be the disruption of the membrane structure and the bacterium cell by the extract, increasing influx of antibiotics inside the bacteria [30]. These actions are generally attributed to some terpenoids [29] and lipophilic flavonoids [31], which can cause a disruption of the plasma membrane of the microorganisms. The extracts or natural compounds can also exert their modulating effects by inhibiting bacterial efflux pumps, allowing an increase of the intracellular concentrations of the antibiotics [24, 32]. RHL has potentiated the activity of TET and KAN on more than 70% of the tested MDR bacteria. This suggests that some compounds of that extract may act as efflux pump inhibitors [33]. View that it is the first time here to report the potential of the *R. heudelotii* leaves extract (RHL) to reverse antibiotic resistance in MDR bacteria, that plant extract could be used for the screening of antibiotic modulators, especially efflux pumps inhibitors.

Various classes of phytochemical compounds were previously found in the tested plant extracts [34]. The most active extract (RHL) contained terpenoids and saponins absent in the bark extract (RHB). This suggests that the pronounced antibacterial activity as well as antibiotic-modulating effects of the leaves extract of *R. heudelotii* could be due to the presence of these metabolites.

## Conclusion

In conclusion, this study have provided informative data about the antimicrobial potential of the tested plant extracts by suggesting that *R. heudelotii* methanol leaves extract could be a source of natural antibacterial products as well as that for antibiotics resistance modulators. This provides a new weapon against the problem of bacterial resistance to antibiotics.

## Abbreviations

AMP: ampicillin
CFU: colony forming unit
CHL: chloramphenicol
CIP: ciprofloxacin
DMSO: Dimethyl-sulfoxide
ERY: erythromycin
INT: *p*-iodonitrotetrazolium chloride
KAN: kanamycin
MBC: minimal bactericidal concentration
MDR: multidrug-resistant
MHB: Mueller Hinton Broth
MIC: minimal inhibitory concentration
PAßN: Phenylalanine arginine *β*-naphthylamide
*R. heudelotii*: *Recinodindron heudelotii*
RHB: *Recinodindron heudelotii* bark extract
RHL: *Recinodindron heudelotii* leaves extract
RND: resistance nodulation cell division
SRF/Cam: *Sociètè des Reserves Forestières du Cameroun*
TET: tetracycline
